# Association of adiponectin with type 2 diabetes and hypertension in African American men and women: the Jackson Heart Study

**DOI:** 10.1186/s12872-015-0005-5

**Published:** 2015-02-25

**Authors:** Sharon K Davis, Samson Y Gebreab, Ruihua Xu, Pia Riestra, Rumana J Khan, Anne E Sumner, DeMarc Hickson, Aurelian Bidulescu

**Affiliations:** National Human Genome Research Institute, Genomics of Metabolic, Cardiovascular and Inflammatory Disease Branch, Social Epidemiology Research Unit, 10 Center Drive, 20892 Bethesda, MD USA; National Institute of Diabetes and Digestive and Kidney Diseases, Section on Ethnicity and Health, 10 Center Drive, 20892 Bethesda, MD USA; Department of Medicine, University of Mississippi Medical Center, 2500 North Street, 39216 Jackson, MS USA; Indiana University Bloomington, School of Public Health, 1025 E. 7th Street, Suite 111, 47405 Bloomington, IN USA

**Keywords:** Biomarkers, Types 2 diabetes, Hypertension, African Americans

## Abstract

**Background:**

Adiponectin is a biomarker that is associated with type 2 diabetes and hypertension. Lower circulating level is a risk factor. Higher levels are protective. African Americans have a higher prevalence of type 2 diabetes and hypertension and lower levels of adiponectin when compared to other racial/ethnic groups. Little is known about the association of adiponectin on these health outcomes among African Americans. The purpose of the study was to assess the association of adiponectin on type 2 diabetes and hypertension likelihood among African American men and women in the Jackson Heart Study.

**Methods:**

Separate multivariate logistic regressions were conducted stratified by sex based on cross-sectional data with type 2 diabetes and hypertension as the outcomes. Adiponectin was divided into four quartiles with the highest quartile as the reference. Data was collected from 2000-2004 on 3,663 participants. Data analysis was conducted in calendar year 2014. Two- tailed *P* < .05 was established as level of significance.

**Results:**

In the adjusted multivariate models, adiponectin level was inversely associated with type 2 diabetes among women (odds ratio [OR], 95% confidence interval [CI] = 1.47, [1.02, 2.11], *P* = .04). There was no association among men. Women with the lowest level of adiponectin were less likely to be hypertensive (OR, 95% CI = 0.66, [0.46, 0.95], *p* = .02). There was no association among men.

**Conclusion:**

Findings reveal differential associations between levels of adiponectin with type 2 diabetes and hypertension likelihood among African American women. More research is needed to elucidate this differential association.

## Background

Obesity is a modifiable risk factor for hypertension and type 2 diabetes. High body mass index (BMI) is a measure of obesity and is associated with an increase in blood pressure and a greater risk of type 2 diabetes and hypertension [1]. Studies have shown that adiponectin has a profound effect on metabolism and vasculature, and is inversely associated with type 2 diabetes and hypertension [2-4]. Lower levels of circulating adiponectin play an important role in the pathogenesis of such conditions [5,2,3]. Circulating adiponectin levels have been shown to be inversely associated with BMI [5]. Laboratory studies suggest that hyperadiponectinemia suppresses several pathophysiological conditions including obesity-related insulin resistance, endothelial dysfunction, and inflammation [6]. Studies show that African Americans have lower circulating adiponectin when compared to other racial/ethnic groups [7]. Studies have also shown that level of adiponectin substantially vary by lifestyle and socioeconomic status (SES) [8,9]. Higher levels of adiponectin has been shown to be protective against obesity-related type 2 diabetes and hypertension. African Americans have lower SES status, a higher prevalence of obesity, type 2 diabetes, hypertension, and poorer lifestyle profiles when compared to other racial/ethnic groups in the United States [10]. The majority of previous studies related to adiponectin have involved Asian or White populations, with few studies in African Americans [2,3,6]. Furthermore, these few studies that included African Americans were limited by smaller sample sizes and did not investigate the association by gender [11,12]. Only two population-based studies with large African American samples were identified. Using the Atherosclerosis Risk in Communities (ARIC) study, Duncan et al found higher adiponectin was associated with lower incidence of types 2 diabetes in African Americans and White participants but did not investigate the association with hypertension [13]. Another study by Wang et al found inverse association between adiponectin and risk of hypertension in African American postmenopausal women but the study did not include men [12]. Many of these studies have also not made adequate adjustment for important confounding factors, such as health behaviors, biomedical factors and SES. Therefore, investigating the association of adiponectin and type 2 diabetes and hypertension in a large sample of economically diverse African Americans will provide additional insight.

Cross-sectional and case-control studies show a significantly higher risk and incidence of type 2 diabetes and hypertension correlated with lower levels of adiponectin in African American men and women [11-13]. Few studies have assessed the differential association of adiponectin on type 2 diabetes and hypertension within African Americans [14-16]. The objective of this study was to investigate the associations of adiponectin with type 2 diabetes and hypertension in a large economically diverse sample of African American men and women using cross-sectional data from the Jackson Heart Study. It is hypothesized that, after adjusting for confounders, including SES, men and women with lower levels of adiponectin would have higher likelihood of type 2 diabetes and hypertension.

## Methods

### Study subjects

The Jackson Heart Study is a single-site, prospective cohort study of risk factors and causes of heart disease in adult African Americans. A probability sample of 5,301 African Americans, aged 21-94 years, residing in three contiguous counties surrounding Jackson, Mississippi, was recruited and examined at baseline from 2000-2004 by certified technicians according to standardized protocols [17,18]. The present study includes cross-sectional data on 3,663 participants who had complete data on all variables of interest. The missing values included 798 for annual household income, 496 for fasting insulin, 100 for C-reactive protein (CRP), 132 for plasma leptin and 174 for circulating plasma adiponectin. The Jackson Heart Study baseline examination included blood pressure, anthropometry, survey of medical history, cardiovascular risk factors and collection of blood and urine for biological variables. Written consent was obtained from each participant at the inception of the study. The study protocol was approved by the Institutional Review Boards of the National Institutes of Health and the participating Jackson Heart Study institutions – including the University of Mississippi Medical Center, Tougaloo College and the University of Mississippi Medical Center. Data analysis for this study was conducted in calendar year 2014.

### Outcome variables

Type 2 diabetes was defined as fasting plasma glucose ≥ 126 mg/dL or self-reported use of insulin or oral hypoglycemic medications [2]. Hypertension was based on a systolic blood pressure (SBP) of ≥ 140 mmHg, diastolic blood pressure (DBP) ≥ 90 mmHg, or self-reported medication use specifically for elevated blood pressure [3]. Blood pressure was measured using standard protocols with participant sitting quietly for 5 minutes measured at 1-minute intervals. The average of two sitting blood pressure was used in the analysis.

### Primary predictor

Adiponectin measurement was derived from venous blood samples drawn from each participant at baseline after more than 8 hours of fasting. Vials of serum were stored at the Jackson Heart Study central repository in Minneapolis, MN, at -80°C until assayed. Adiponectin concentration was measure in 2008-2012 as total circulating plasma adiponectin by an ELISA system (R&D Systems; Minneapolis, MD) [17]. The inter-assay coefficient of variation was 8.8%. No biological degrading has been described using stored specimens, indicating a high validity for measurement [19].

### Covariates

All covariate variables were collected at baseline and were chosen because they are known risk factors for type 2 diabetes and hypertension [10]. Age was derived from date-of-birth. Socioeconomic status was based on self-reported annual household income and divided into three categories (≤ $19,999, $20,000 - $49,999, ≥ $50,000). Biological risk factor measures included fasting insulin, low-density lipoprotein cholesterol (LDL), high-density lipoprotein cholesterol (HDL), total cholesterol (TC), homeostasis model assessment –insulin resistance (HOMA-IR), CRPand plasma leptin. Behavioral risk factor variables included smoking status, physical activity, alcohol status, and body mass index (BMI). Fasting insulin, LDL, HDL, and TC were assessed using standard laboratory techniques. Insulin resistance status was estimated with the HOMA as insulin [20]. CRP was measured using immunoturbidimetric CRP-Latex assay from Kamiya Biomedical Company following manufacturer’s high-sensitivity protocol [16]. The inter-assay coefficients of variation on control samples repeated in each assay were 4.5 and 4.4% at CRP concentration of 0.45 and 1.56 mg/dL respectively. The reliability coefficient for masked quality-control replicates was 0.95 for the CRP assay. Leptin was collected via venous blood samples drawn from each participant after more than 8 hours of fasting and was analyzed with Human Leptin PIA kit (LINCO Research, St. Charles, MI, USA) [21]. Acceptable coefficient of variation was 10% [21]. Smoking status was defined as current smokers and non-smokers. Physical activity was assessed with a physical activity survey instrument comprised of 4 domains (active living, work, home and garden, and sport and exercise indexes). A total score was the sum of these domains with a maximum of 24. A higher score indicates a higher level of total physical activity. Alcohol consumption status was defined as “yes” if participant reported ever consuming alcohol and “no” for those reporting never consuming alcohol. Body mass index was based on standing height and weight measured in lightweight clothing without shoes or constricting garments and calculated as weight in kilograms divided by height in meters squared (kg/m^2^).

### Statistical analysis

All analyses were stratified by sex because of the differential levels of adiponectin between men and women [14-16]. Descriptive analyses were performed using two sample student t-test for continuous variables including means for age, SBP, DBP, physical activity score, BMI, fasting insulin, LDL, HDL, TC, HOMA-IR, fasting glucose, CRP, plasma leptin, and circulating plasma adiponectin. Chi-square test was used for categorical variables including annual household income (i.e. SES), hypertension status, type 2 diabetes status, smoking status, alcohol consumption status, and circulating plasma adiponectin in 4 quartiles ( 1 = ≤2.70 ug/mL, 2 = >2.70 – ≤4.2 ug/mL, 3 = >4.2 - ≤6.7 ug/mL, 4 = > 6.7 ug/mL). Continuous variables were measured with one-way ANOVA. Age, SES, biological and behavioral variables were assessed according to the four quartiles of circulating plasma adiponectin by sex.

Separate logistic regression models for type 2 diabetes and hypertension were stratified by sex. Five models were analyzed with adiponectin as the primary predictor with quartile 4 representing the highest level of adiponectin as the reference. Model 1 included adiponectin as the primary predictor, model 2 included age, model 3 included biological indices, model 4 included behavioral indices, and model 5 included a fully adjusted model with SES (≥ $50,000 as the reference).

A two-tailed level of significance was established as *P* < .05. Analyses were conducted using SAS version 9.3 [22].

## Results

The sex-stratified baseline characteristics are presented in Table 1. Women were older and had a higher proportion of annual household income ≤ $19,999, $20,000 - $49,999, but a lower proportion of annual household income ≥ $50,000 (*P <* .0001). There was also a significantly higher proportion of women who were hypertensive when compared to men (62% versus 58%, *P* = .008). However, women had significantly lower SBP and DBP when compared to men (*P* < .0001). Sixteen percent of women had type 2 diabetes compared to 13% among men (*P* = .003). A significantly higher proportion of men were current smokers, had higher mean physical activity score, and were current alcohol consumers when compared to women (*P* < .0001, respectively). Women had higher mean BMI than men (*P* < .0001). Women had lower mean LDL cholesterol (*P* = .006), but higher HDL cholesterol (*P* < .0001) and TC (*P* = .005). Men had lower mean HOMA-IR, (*P* = .0002), CRP (*P* < .0001), plasma leptin (*P* < .0001), and circulating plasma adiponectin (*P* < .0001). There were also significantly different proportional distributions between men and women across all 4 quartiles of circulating plasma adiponectin (*P* < .0001).

**Table 1:** **Characteristics of study participants (N = 3,663)**

	**Men (N = 1,334, 36.4%)**	**Women (N = 2,329, 63.6%)**	***P*** **-value** ^**a**^
Age (years), mean ± std^b^	53.4 ± 13.0	54.5 ± 12.7	**.01**
Annual household income,%			
Less than $19,999	19.9	32.3	**<.0001**
$20,000 - 49,999	32.9	39.2	
$50,000 or more	47.2	28.6	
Hypertensive, %	57.7	62.1	**.008**
Systolic blood pressure (mmHg)	127.8 ± 18.1	125.9 ± 18.2	**.002**
Diastolic blood pressure (mmHg)	81.6 ± 10.4	77.5 ± 10.0	**<.0001**
Type 2 Diabetic, %	12.6	16.2	**.003**
Current Smoker,%	16.6	10.4	**<.0001**
Physical activity score	8.8 ± 2.6	8.3 ± 2.5	**<.0001**
Alcohol consumer, %	61.7	40.0	**<.0001**
BMI^c^ (kg/m^2^)	29.8 ± 6.2	32.8 ± 7.6	**<.0001**
Fasting insulin (uU/mL)	17.1 ± 30.7	18.8 ± 15.5	.05
LDL-Cholesterol^d^ (mg/dL)	128.5 ± 36.6	125.1 ± 36.1	**.006**
HDL-Cholesterol^e^ (mg/dL)	46.0 ± 12.5	55.1 ± 14.4	**<.0001**
Total Cholesterol (mg/dL)	196.1 ± 39.1	199.8 ± 39.4	**.005**
HOMA-IR^f^	3.45 ± 2.42	3.78 ± 2.41	**.0002**
Fasting Glucose (mg/dl)	99.4 ± 31.4	99.1 ± 30.9	.78
CRP^g^ (mg/dL)	.37 ± 1.09	.59 ± .84	**<.0001**
Plasma leptin (ng/mL)	11.2 ± 10.6	37.5 ± 23.3	**<.0001**
Circulating Plasma adiponectin (ug/mL)	4.1 ± 3.3	6.0 ± 4.6	**<.0001**
Circulating Plasma adiponectin, **%**			
≤2.70 ug/mL	38.8	17.5	**<.0001**
>2.70 - ≤4.2 ug/mL	26.8	23.8	
>4.2 - ≤6.7 ug/mL	20.3	28.0	
>6.7 ug/mL	14.1	30.7	

^a^Two sample t-test for continuous variables and chi-square for categorical variables; significance established as *P* < .05.

^b^std = standard deviation. All continuous variables present mean ± deviation.

^c^BMI = body mass index.

^d^LDL = low-density lipoprotein.

^e^HDL = high-density lipoprotein.

^f^HOMA-IR = homeostasis model assessment – insulin resistance.

^g^CRP = C-reactive protein.

Table 2 presents the characteristics for men according adiponectin quartiles. There is a significant difference in age across the 4 quartiles, with quartile 4 having the highest mean age of 58 years (*P* < .0001). There are also significant differences in the levels of adiponectin by SES with those with ≥ $50,000 having higher proportions of men in quartile 1, 2, 3, and 4 (*P* < .0001). Mean SBP across the 4 quartiles was higher among men in quartile 4 (*P* < .0001). Mean DBP was highest among men with the lowest level of adiponectin, i.e. quartile 1 (*P* = .04). Mean physical activity score, BMI, fasting insulin, and LDL cholesterol were higher among men in quartile 1 than the other three quartiles (*P* = .007, <.0001, .006, .01, respectively). Mean HDL cholesterol was highest among those with the highest level of adiponectin in quartile 4 (*P* < .0001). Mean HOMA-IR, fasting glucose, and plasma leptin was higher among those in quartile 1 (*P* < .0001, .009, <.0001, respectively).

**Table 2:** **Characteristics among men by circulating plasma adiponectin (N = 1,334)**

	**Plasma Adiponectin (ug/mL)** ^**a**^				
	**Q1(n = 518)**	**Q2(n = 357)**	**Q3(n = 271)**	**Q4(n = 188)**	***P*** **–value** ^**b**^
Age (years), mean ± std^c^	51.07 ± 11.76	53.27 ± 13.04	54.76 ± 13.10	58.28 ± 14.61	**<.0001**
Annual household income,%					
Less than $19,999	13.51	21.01	22.88	30.85	
$20,000 - 49,999	32.82	33.33	33.58	31.38	
$50,000 or more	53.67	45.66	43.54	37.77	**<.0001**
Hypertensive, %	55.98	56.30	57.93	64.89	.18
Systolic blood pressure (mmHg) , mean ± std	126.43 ± 15.88	127.32 ± 18.41	126.87 ± 17.25	133.64 ± 22.81	**<.0001**
Diastolic blood pressure (mmHg), mean ± std	82.57 ± 10.02	81.44 ± 10.39	80.59 ± 10.35	80.80 ± 11.20	**.04**
Type 2 Diabetic, %	12.93	11.48	11.44	15.43	.54
Current Smoker,%	15.06	16.81	16.97	19.68	.53
Physical activity score, mean ± std	8.98 ± 2.49	8.80 ± 2.67	8.77 ± 2.51	8.21 ± 2.75	**.007**
Alcohol consumption, %	64.67	59.38	61.99	57.45	.24
BMI^d^ (kg/m^2^), mean ± std	31.06 ± 6.21	29.85 ± 6.14	29.38 ± 6.17	26.76 ± 5.51	**<.0001**
Fasting insulin (uU/mL), mean ± std	20.71 ± 44.12	15.77 ± 11.06	14.65 ± 9.99	13.31 ± 30.31	**.006**
LDL^e^ Cholesterol (mg/dL), mean ± std	131.27 ± 37.67	128.19 ± 36.35	128.50 ± 34.80	121.19 ± 35.63	**.01**
HDL^f^ Cholesterol (mg/dL), mean ± std	42.13 ± 10.24	44.43 ± 10.30	48.45 ± 11.32	56.21 ± 16.79	**<.0001**
Total Cholesterol (mg/dL), mean ± std	5.09 ± 1.04	4.97 ± 1.00	5.03 ± 0.95	4.97 ± 0.97	.31
HOMA-IR^g^, mean ± std	4.17 ± 2.97	3.28 ± 1.89	3.06 ± 1.97	2.30 ± 1.39	**<.0001**
Fasting Glucose (mg/dL), mean ± std	102.24 ± 33.53	100.01 ± 36.59	96.52 ± 25.07	94.34 ± 20.08	**.009**
CRP^h^ (mg/dL),mean ± std	0.43 ± 1.59	0.36 ± 0.63	0.32 ± 0.49	0.30 ± 0.65	.40
Plasma leptin (ng/mL), mean ± std	12.28 ± 10.77	11.06 ± 9.47	11.90 ± 12.57	7.82 ± 8.38	**<.0001**

^a^Adiponectin in quartiles (Q = quartile): 1) Q1 = ≤2.70 ug/mL, 2) Q2 = >2.70 – ≤4.2 ug/mL, 3)Q3 = >4.2 – ≤6.7 ug/mL, 4) Q4 > 6.7 ug/mL.

^b^One-way ANOVA for continuous variables and chi-square for categorical variables; significance established as *P* < .05.

^c^std = standard deviation.

^d^BMI = body mass index.

^e^LDL = low-density lipoprotein.

^f^HDL = high-density lipoprotein.

^g^HOMA-IR = homoeostasis model assessment – insulin resistance.

^h^CRP = C-reactive protein.

Table 3 presents the characteristics for women according to the 4 quartiles of adiponectin. As revealed in men, higher mean age for women were in quartile 4 (57 years, *P* < .0001). Women with higher mean SBP also had the highest levels of adiponectin in quartile 4 (*P* < .0001). A higher proportion of women who had type 2 diabetes had the lowest level of adiponectin in quartile 1(*P* = .0002). Women with higher mean BMI and fasting insulin also had the lowest level of adiponectin in quartile 1(*P* < .0001, <.0001, respectively). Those with higher mean HDL cholesterol and TC had the highest level of adiponectin in quartile 4 (*P* < .0001, .04, respectively). Women with higher mean HOMA-IR, fasting glucose, CRP, and plasma leptin have the lowest level of adiponectin in quartile 1(*P* < .0001, .0008, <.0001, .0001).

**Table 3:** **Characteristics among women by circulating plasma adiponectin (N = 2,329)**

	**Plasma Adiponectin(ug/mL)** ^**a**^				
	**Q1(n = 408)**	**Q2(n = 554)**	**Q3(n = 652)**	**Q4(n = 715)**	***P*** **-value** ^**b**^
Age (years), mean ± std^c^	51.94 ± 12.17	52.55 ± 11.85	55.08 ± 12.74	57.10 ± 12.93	**<.0001**
Annual household income,%					
Less than $19,999	36.03	30.32	29.75	33.85	
$20,000 - 49,999	37.01	39.89	43.56	35.80	
$50,000 or more	26.96	29.78	26.69	30.35	.05
Hypertensive, %	61.03	61.01	62.27	63.50	.78
Systolic blood pressure (mmHg) , mean ± std	124.48 ± 18.47	123.63 ± 15.85	126.02 ± 18.89	128.26 ± 18.82	**<.0001**
Diastolic blood pressure (mmHg), mean ± std	76.97 ± 9.56	77.50 ± 10.06	77.55 ± 9.70	77.65 ± 10.54	.73
Type 2 Diabetic, %	22.30	16.25	13.96	14.69	**.0002**
Current Smoker,%	13.73	10.65	9.51	9.09	.08
Physical activity score, mean ± std	8.52 ± 2.45	8.49 ± 2.45	8.20 ± 2.50	8.21 ± 2.60	**.04**
Alcohol consumption, %	40.20	43.32	38.96	38.32	.30
BMI^d^ (kg/m^2^), mean ± std	34.63 ± 7.37	34.01 ± 6.81	32.80 ± 7.65	30.94 ± 7.70	**<.0001**
Fasting insulin (uU/mL), mean ± std	26.69 ± 17.56	21.05 ± 20.03	17.42 ± 11.78	13.88 ± 10.30	**<.0001**
LDL^e^ Cholesterol (mg/dL), mean ± std	124.67 ± 36.56	126.47 ± 36.39	124.91 ± 35.04	124.33 ± 36.76	.76
HDL^f^ Cholesterol (mg/dL), mean ± std	48.01 ± 11.89	51.49 ± 11.73	55.65 ± 13.15	61.53 ± 15.90	**<.0001**
Total Cholesterol (mg/dL), mean ± std	5.03 ± 1.00	5.10 ± 1.01	5.12 ± 0.98	5.20 ± 1.04	**.04**
HOMA-IR^g^, mean ± std	5.28 ± 2.91	4.28 ± 2.45	3.65 ± 2.37	2.73 ± 1.40	**<.0001**
Fasting Glucose (mg/dL), mean ± std	103.64 ± 31.30	100.78 ± 31.50	97.22 ± 28.14	96.84 ± 32.31	**.0008**
CRP^h^ (mg/dL),mean ± std	0.73 ± 0.91	0.72 ± 0.95	0.61 ± 0.85	0.40 ± 0.63	**<.0001**
Plasma leptin (ng/mL), mean ± std	38.64 ± 19.28	40.18 ± 20.66	37.90 ± 23.81	34.43 ± 26.47	**.0001**

^a^Adiponectin in quartiles (Q = quartile): 1) Q1 = ≤2.70 ug/mL, 2) Q2 = >2.70 – ≤4.2 ug/mL, 3)Q3 = >4.2 – ≤6.7 ug/mL, 4) Q4 > 6.7 ug/mL.

^b^One-way ANOVA for continuous variables and chi-square for categorical variables; significance established as *P* < .05.

^c^std = standard deviation.

^d^BMI = body mass index.

^e^LDL = low-density lipoprotein.

^f^HDL = high-density lipoprotein.

^g^HOMA-IR = homoeostasis model assessment – insulin resistance.

^h^CRP = C-reactive protein.

### Multivariable regression models with adiponectin on type 2 diabetes and hypertension

Table 4 reveals there were no significant associations between adiponectin and type 2 diabetes in the crude and multivariable models among men. Although non-significant, those with the lowest level of adiponectin (quartile 1) had higher likelihood of having type 2 diabetes (odds ratio [OR], 95% confidence interval [CI] = 1.12,[0.68,1.83], *P* = .65) in the age adjusted model. This association attenuated and remained non-significant in a fully adjusted model [OR, 95%CI =0.75, [0.43,1.30], *P* = .30]. In the crude model, women with the lowest level of adiponectin were associated with a higher likelihood (67%) of having type 2 diabetes when compared to those with the highest level of adiponectin (OR, 95%CI = 1.67, [1.22, 2.28], *P* = .001). The model adjusted for age revealed those with lowest and lower levels of adiponectin (i.e. quartile 1 and 2) were twice and 1.42 times more likely to have type 2 diabetes compared to women with the highest level of adiponectin (quartile 4) (95% CI = [1.58, 3.03], [1.03, 1.95], *P* < .0001 and .04, respectively). The model adjusted for biological risk factors revealed women with the lowest level of adiponectin were 47% more likely to have type 2 diabetes than women with the highest level ( OR, 95% CI = 1.47, [1.02, 2.12], *P* = .03). The model adjusted for behavioral risk factors similarly revealed that women with the lowest and lower levels of adiponectin were 2.19 and 1.41 times more likely to have type 2 diabetes than those with the highest level (95% CI = [1.58, 3.03], [1.03, 1.94], *P* < .0001 and .03, respectively). The fully adjusted model with SES included reveal women with the lowest level of adiponectin retained a significant association with type 2 diabetes (OR, 95% CI = 1.47, [1.02, 2.11], *P* = .04).

**Table 4:** **Association between adiponectin** ^**a**^ **and type 2 diabetes using crude and adjusted logistic regression among men and women (N = 3,663)**

	**Men (N = 1,334)**		**Women (N = 2,329)**	
**Predictor**	**Odds ratio (95% confidence interval)**	***P*** **-value**	**Odds ratio (95% confidence interval)**	***P*** **-value**
Adiponecin Q1^b^	0.81 (0.51, 1.31)	.39	1.67 (1.22, 2.28)	**.001**
Adiponectin Q2	0.71 (0.43, 1.19)	.19	1.13 (0.83, 1.53)	.44
Adiponectin Q3	0.71 (0.41, 1.22)	.21	0.94 (0.69, 1.28)	.70
Q4	1.0		1.0	
Adiponectin Q1^c^	1.12 (0.68, 1.83)	.65	2.19 (1.58, 3.03)	**<.0001**
Adiponectin Q2	0.88 (0.52, 1.48)	.62	1.42 (1.03, 1.95)	**.04**
Adiponectin Q3	0.82 (0.47, 1.43)	.49	1.03 (0.76, 1.41)	.83
Q4	1.0		1.0	
Adiponectin Q1^d^	0.70 (0.40, 1.21)	.20	1.47 (1.02, 2.12)	**.03**
Adiponectin Q2	0.64 (0.37, 1.12)	.12	1.10 (0.79, 1.55)	.58
Adiponectin Q3	0.64 (0.36, 1.14)	.13	0.86 (0.62, 1.20)	.38
Q4	1.0		1.0	
Adiponectin Q1^e^	1.13 (0.69, 1.85)	.63	2.19 (1.58, 3.03)	**<.0001**
Adiponectin Q2	0.87 (0.52, 1.48)	.61	1.41 (1.03, 1.94)	**.03**
Adiponectin Q3	0.83 (0.48, 1.45)	.51	1.02 (0.75, 1.39)	.90
Q4	1.0		1.0	
Adiponectin Q1^f^	0.75 (0.43, 1.30)	.30	1.47 (1.02, 2.11)	**.04**
Adiponectin Q2	0.65 (0.37, 1.14)	.13	1.10 (0.78, 1.55)	.58
Adiponectin Q3	0.64 (0.36, 1.16)	.14	0.86 (0.62, 1.22)	.36
Q4	1.0		1.0	

^a^Adiponectin in quartiles (Q = quartile): 1) Q1 = ≤2.70 ug/mL, 2) Q2 = >2.70 – ≤4.2 ug/mL, 3)Q3 = >4.2 – ≤6.7 ug/mL, 4) Q4 > 6.7 ug/mL. The referent group is Q4 > 6.7 ug/mL.

^b^Model 1, crude.

^c^Model 2, partly adjusted for age.

^d^Model 3, partly adjusted for age, low-density lipoprotein cholesterol, high-density lipoprotein cholesterol, total cholesterol, c-reactive protein, plasma leptin.

^e^Model 4, partly adjusted for age, body mass index, smoking status, physical activity score and alcohol consumption status.

^f^Model 5, fully adjusted for age, body mass index, low-density lipoprotein cholesterol, high-density lipoprotein cholesterol, total cholesterol, plasma leptin, C-reactive protein, smoking status, physical activity score, alcohol consumption status and socioeconomic status based on annual household income.

A two-tailed level of significance was established as *P* < .05.

Table 5 presents the findings for the association of adiponectin and hypertension. In the age-adjusted model, men with the lowest level of adiponectin were associated with non-significant increase in the prevalence of hypertension (OR, 95% CI = 1.07, [0.73, 1.58], *P* = .72). The associations remained non-significant in the subsequent multivariate models. In the model adjusted for age, women with the lowest and lower levels of adiponectin (quartile 1 and 2) were significantly more likely to have hypertension (OR, 95% CI = 1.39, 1.32 [1.05, 1.85], [1.02, 1.70], *P* = .02, .04, respectively). In the models adjusted for biological and behavioral risk factors, women with the lowest level of adiponectin were 33% less likely to be hypertensive when compared to women with the highest level of adiponectin (OR, 95% CI = 0.67, [0.46, 0.96], *P* = .03) and 34% (OR, 95% CI = 0.67, [0.46, 0.96], *P* = .03), respectively. After final adjustments for SES, women with the lowest level of adiponectin remained significantly less likely to be hypertensive then those with the highest level (OR, 95% CI = 0.66, [0.46, 0.95], *P* = .02).

**Table 5:** **Association between adiponectin** ^**a**^ **and hypertension using crude and adjusted logistic regression among men and women (N = 3,663)**

	**Men (N = 1,334)**		**Women (N = 2,329)**	
**Predictor**	**Odds ratio (95% confidence interval)**	***P*** **-value**	**Odds ratio (95% confidence interval)**	***P*** **-value**
Adiponectin Q1^b^	0.69 (0.49, 0.97)	**.03**	0.90 (0.70, 1.16)	.41
Adiponectin Q2	0.70 (0.48, 1.00)	.05	0.90 (0.72, 1.13)	.36
Adiponectin Q3	0.75 (0.51, 1.10)	.13	0.95 (0.76, 1.18)	.64
Q4	1.0		1.0	
Adiponectin Q1^c^	1.07 (0.73, 1.58)	.72	1.39 (1.05, 1.85)	**.02**
Adiponectin Q2	0.94 (0.63, 1.41)	.76	1.32 (1.02, 1.70)	**.04**
Adiponectin Q3	0.92 (0.60, 1.40)	.68	1.13 (0.88, 1.45)	.34
Q4	1.0		1.0	
Adiponectin Q1^d^	0.79 (0.49, 1.26)	.32	0.67 (0.46, 0.96)	**.03**
Adiponectin Q2	0.75 (0.47, 1.20)	.23	0.86 (0.63, 1.16)	.32
Adiponectin Q3	0.85 (0.53, 1.37)	.51	0.86 (0.65, 1.13)	.28
Q4	1.0		1.0	
Adiponectin Q1^e^	0.79 (0.49, 1.26)	.33	0.66 (0.46, 0.95)	**.03**
Adiponectin Q2	0.75 (0.47, 1.19)	.22	0.85 (0.63, 1.16)	.33
Adiponectin Q3	0.85 (0.53, 1.37)	.51	0.85 (0.64, 1.13)	.28
Q4	1.0		1.0	
Adiponectin Q1^f^	0.80 (0.50, 1.28)	.36	0.66 (0.46, 0.95)	**.02**
Adiponectin Q2	0.75 (0.47, 1.20)	.23	0.86 (0.63, 1.16)	.32
Adiponectin Q3	0.86 (0.53, 1.38)	.52	0.85 (0.64, 1.12)	.25
Q4	1.0		1.0	

^a^Adiponectin in quartiles (Q = quartile): 1) Q1 = ≤2.70 ug/mL, 2) Q2 = >2.70 – ≤4.2 ug/mL, 3)Q3 = >4.2 – ≤ 6.7 ug/mL, 4) Q4 > 6.7 ug/mL. The referent group is Q4 > 6.7 ug/mL.

^b^ = Model 1, crude.

^c^ = Model 2, partly adjusted for age.

^d^ = Model 3, partly adjusted for age, fasting insulin**,** HOMA-IR(Insulin resistance), low-density lipoprotein cholesterol, high-density lipoprotein cholesterol, total cholesterol, c-reactive protein, plasma leptin.

^e^ = Model 4, partly adjusted for age, body mass index, smoking status, physical activity score, alcohol consumption status.

^f^ = Model 5, fully adjusted for age, body mass index, low-density lipoprotein cholesterol, high-density lipoprotein cholesterol, total cholesterol, plasma leptin, fasting insulin, HOMA-IR, C-reactive protein, smoking status, physical activity score, alcohol consumption status, and socioeconomic status based on annual household income.

A two-tailed level of significance was established as *P* < .05.

## Discussion

This study was carried out in a large sample of community-based middle to older aged African American men and women. We found that lower adiponectin level was associated with higher likelihood of having type 2 diabetes, but with a lower likelihood of having hypertension in African American women. However, the associations of adiponectin with type2 diabetes and hypertension were less consistent in African American men.

Few studies have assessed the association of adiponectin on type 2 diabetes and hypertension risk in African Americans [11-13]. Our findings of inverse association between adiponectin levels and type 2 diabetes among women are consistent with several previous studies, although in men this association was less consistent. For instance, Duncan et al analysis of the effects of adiponectin on risk of type 2 diabetes among a large community based sample revealed African Americans with lower quartiles of adiponectin had significantly higher risk of type 2 diabetes after multiple adjustments for confounders [13]. Hanley et al, likewise, demonstrated a significant inverse relationship between adiponectin and incidence of type 2 diabetes among African Americans [11]. Conversely, a cohort study of older African American participants showed no significant association between level of adiponectin and incidence of type 2 diabetes [23]. The reasons for the sex-specific difference in the association of adiponectin with type 2 diabetes are and hypertension is unclear. However, it may be attributable to gender differences in adiposity. African American women in this study tend to have a higher BMI, WC and percentage of body fat, especially subcutaneous fat, than African American men [15]. In addition, it is also possible sex-specific hormones, such as estradiol and testosterone, may also explain the sex difference in the association of adiponectin with diabetes and hypertension in our study [24-26]. Further research on adiponectin and type 2 diabetes and hypertension on both sexes accounting for sex hormones and adiposity measures is warranted to elucidate the biological mechanisms of this association.

Findings regarding hypertension demonstrated women with lower quartiles of adiponectin were less likely to be hypertensive when compared to those in the highest quartile. Although not significant, we also observed similar pattern in men. These ‘paradoxical’ findings between adiponectin and hypertension are inconsistent with several previous studies that reported inverse association between adiponectin and hypertension [3,11,12]. For example, Wang et al reported a significant inverse relationship between adiponectin and hypertension in African American women [12]. Iwashima et al likewise reported a significant association between low levels of adiponectin and hypertension in a Japanese study population [3]. However, other studies have also reported similar findings [14,27-30]. One animal study in which rats were feed a high salt diet exhibited hypertension associated with elevated levels of adiponectin [28]. Increased levels of adiponectin have been described in hospital patients with both systolic and diastolic forms of heart failure [29]. One of the authors in the current population study found significant association of increased adiponectin among hypertensives with left ventricular mass among African Americans [14]. Similarly, in the Health ABC biracial cohort, higher circulating concentrations of adiponectin were associated with higher risk of coronary heart disease in older African Americans, even after adjustments for known risk factors [23]. The biological underlying of such “paradoxical” association between adiponectin and hypertension is not clear. However, it has been suggested that the beneficial effects of adiponectin may depend on other factors involved in blood pressure regulation, such as excess dietary salt intake, activation of the renin-angiotensin-aldosterone system as well as metabolic factors [3-6]. Although several studies report an inverse protective association between adiponectin levels and type 2 diabetes and hypertension there are other investigations that indicate the opposite [3,5,6,11-14,23,27-30].

These discrepancies are probably due to the different characteristics in the study populations. This study revealed higher levels of adiponectin was protective for the probability of type 2 diabetes and lower levels was protective for hypertension among women. Findings differ from other studies which show similar magnitude of association between men and women [11]. In addition, adiponectin resistance has been posited to explain some of the “paradoxical” associations of adiponectin with adverse outcomes [31,32].

Findings regarding hypertension demonstrated men and women with lower quartiles of adiponectin were less likely to be hypertensive when compared to those in the highest quartile and therefore indeed may be indicative of adiponectin resistance. Findings are inconsistent with other investigations that showed a significant inverse association between adiponectin and hypertension likelihood [3,12,13,33]. However, such studies assessed one gender and did not include both genders. Only one study compared the association between White and African Americans [13]. Adiponectin could serve as a marker of disease severity in older adults with risk factors but not to the same degree as younger individuals. In addition to its protective effects, increased levels of adiponectin may be harmful particularly among the elderly. This is demonstrated in this study as observed among women when age was included as a continuous variable in the models adjusted for biological, behavioral, and SES factors.

### Strengths and limitations

The strength of this investigation is that findings were from the largest community-based sample of African Americans, a cohort with strict protocol and high quality-control. It also addresses two of the most important health outcomes that disproportionately affect African Americans. In addition, it presents differential findings among African American women and men regarding the association of adiponectin with type 2 diabetes and hypertension. One limitation of the study is that findings cannot be generalizable to other ethnic groups. Secondly, this is a cross-sectional analysis, thus, we cannot establish causal relationships between adiponectin and type 2 diabetes and hypertension. Furthermore, residual confounding may have impacted the results. Although we adjusted for several known confounders, our study did not adjust for other confounders, such as dietary salt intake, sex hormones, and adiposity measures. Finally, our study used total adiponectin rather high molecular weight (HMW) adiponectin , which is considered the most biologically active form. This could also potentially affect our findings since some studies suggested that difference in biological activity between different isoforms of adiponectin and metabolic abnormalities [34], but another study has also demonstrated that HMW does not provide more significant information than total adiponectin [35].

## Conclusion

The major finding of this study was that after adjustments for age, biology, behavior and SES, adiponectin was inversely associated with type 2 diabetes in African American women, but the association was less consistency in men. Furthermore, we also found that women with lower levels of adiponectin were less likely to be hypertensive. More research is needed to elucidate the differential associations of adiponectin with type 2 diabetes and hypertension among African American women and men.
